# A Life Without Olfactory Function Has Limited Effects on Cerebral White Matter Morphology

**DOI:** 10.1155/np/3960593

**Published:** 2026-05-16

**Authors:** Anja L. Winter, Moa Peter, Evelina Thunell, Sanne Boesveldt, Johan N. Lundström, Fahimeh Darki

**Affiliations:** ^1^ Department of Clinical Neuroscience, Karolinska Institutet, Stockholm, Sweden, ki.se; ^2^ Division of Human Nutrition and Health, Wageningen University and Research, Wageningen, Gelderland, Netherlands, wur.nl; ^3^ Department of Otorhinolaryngology, Karolinska University Hospital, Stockholm, Sweden, karolinska.se; ^4^ Monell Chemical Senses Center, Philadelphia, Pennsylvania, USA, monell.org

**Keywords:** MRI, olfaction, sensory loss, smell

## Abstract

Absence of sensory input is associated with alterations in brain morphology; mainly in or near cerebral regions normally devoted to processing of the missing sense. However, multiple recent studies demonstrate that the only consistent morphological finding within the gray matter of individuals born without the sense of smell (isolated congenital anosmia [ICA]), are changes in or near the olfactory sulcus. In contrast, for the connecting tissue of the brain, the white matter (WM), previous studies have yielded inconsistent findings. Here, we show that individuals with ICA (*n* = 49) exhibit clear alterations in WM volume when compared to age‐ and sex‐matched controls. Consistent evidence from both voxel‐based morphometry and multivoxel pattern analysis shows that individuals with ICA show decreased WM in areas surrounding the olfactory sulcus. Critically, no WM alterations were found in areas surrounding the olfactory (piriform) cortex. In contrast to congenital sensory loss in other sensory systems, we show that morphological alterations due to lifelong olfactory deprivation are limited, primarily localized around the olfactory sulcus, and likely due to the absence of olfactory bulbs. A possible explanation for the lack of major morphological alterations in individuals with congenital anosmia is that the olfactory regions may be recruited for nonolfactory functions.

## 1. Introduction

Sensory deprivation, an extended and significant reduction in (or complete absence of) sensory input, has commonly been associated with altered brain morphology in areas that typically process information from the absent sense [1–3]. These morphological changes are especially pronounced in cases of lifelong deprivation where gray matter changes are present in several processing areas [4–6]. Adults who were born without a sense of smell with no other underlying medical conditions (isolated congenital anosmia [ICA]) display morphological deviations in the orbitofrontal cortex (OFC) [2, 7], which has been described as the primary area of advanced olfactory processing [8, 9]. Interestingly, these changes occur mainly outside of the primary olfactory cortex [2], with gray matter volume atrophy in the bilateral olfactory sulci and increased gray matter volume in the medial orbital gyri; and no apparent changes in the cortical structure most associated with olfactory processing, the piriform cortex. Previous studies have focused mainly on gray matter morphology (the processing dimension), and significantly less work has been devoted to assessing white matter (WM) morphology (the communication dimension); we therefore know substantially less about how olfactory deprivation affects the deeper tissues of the brain.

The effects of sensory loss on WM have been established in vision and audition, with similar findings of changes in volume in both modalities [6, 10]. For instance, congenitally blind individuals express overall a reduction of WM volume compared to healthy controls, with specific alterations in areas necessary for the transfer of visual information between the two hemispheres [11]. WM changes are also prominent in individuals with acquired vision loss, who show more extensive alterations compared to congenitally blind individuals [12]. Similarly, deaf individuals exhibit alterations in WM structural parameters, such as reductions in volume [3], density [13], and fractional anisotropy [14]. Not surprisingly, these alterations occur across both the primary and secondary auditory cortices, mainly in the bilateral superior temporal gyrus [4]. In essence, various studies on visual and auditory deprivation have established an effect on WM morphology with possible variation depending on the extent and origin of the loss. Yet, the putative effects of olfactory deprivation on WM morphology remain largely unexplored.

There have been reports of decreased WM volume in secondary olfactory areas in individuals with anosmia [15], particularly in cases of acquired olfactory loss [16] resulting from conditions such as COVID‐19 [17] or trauma [18]. Furthermore, a positive correlation has been observed between the duration of anosmia and the extent of WM atrophy [15]. However, a few studies have reported a larger volume [16], increased density [16, 19], and heightened fractional anisotropy of WM among individuals with ICA. Previous studies on brain morphology in anosmia, particularly congenital anosmia, report disparate effects in primary olfactory areas such as the piriform cortex. These inconsistencies may be attributed to variability in the duration and etiology of anosmia, as such diversity, both within and across studies, makes it difficult to draw definitive conclusions about the condition. In addition, many studies show weak effects that might be explained by their small sample sizes.

Here, we assessed alterations in WM morphology in a comparably large cohort of individuals who were born without the sense of smell, compared to a group of healthy controls. In this very same sample, we previously found increased gray matter volume in the bilateral medial orbital gyrus and decreased gray matter volume in the bilateral olfactory sulcus compared to healthy controls [2, 7], and we therefore hypothesized that ICA individuals would demonstrate WM alterations in areas linking these regions. Moreover, we previously found no significant difference in gray matter volume in the piriform cortex of individuals with ICA compared to healthy controls. This might seem surprising considering the lifelong absence of olfactory input but may be partially explained by increases in visual and auditory processing within the region. Based on this, we expected to find an altered WM volume adjacent to the piriform cortex in individuals with ICA as compared to normosmic controls.

## 2. Materials and Methods

### 2.1. Participants

A total of 98 participants were included in the study, 49 individuals with ICA and 49 controls, matched in terms of sex and age (Table 1). For the ICA group, the inclusion criterion was a self‐reported lifelong lack of smell perception without a known underlying condition. The lack of smell perception was confirmed by the olfactory assessment described below. For the control group, the inclusion criterion was a normal sense of smell confirmed by the same olfactory assessment. Out of the 49 individuals in the ICA group, 37 lacked olfactory bulbs bilaterally. The presence of olfactory bulbs in the remaining 12 was nondeterminable in that it was not clear if the potential finding was a unilateral hypoplastic olfactory bulb or image artifact (assessed from structural images). Data were collected in two countries, Sweden (*n* = 38 ICA, 38 controls) and the Netherlands (*n* = 11 ICA, 11 controls). All participants provided written informed consent prior to participation, and the study was approved by ethical review boards in both Sweden (2015/1585‐31) and the Netherlands (NL69840.081.19). A preprint of this study has previously been published [20].

**Table 1:** Descriptive statistics of participants per group.

	ICA (*n* = 49)	Control (*n* = 49)
Sex		
Male	20	20
Female	29	29
Age (years)	36.3 ± 11.7 (20–64)	36.1 ± 11.5 (20–62)
TDI	10.3 ± 2.4 (6–15)	35.5 ± 3.5 (27.75–45)

*Note:* Values within parentheses denote range.

### 2.2. Procedure

#### 2.2.1. Olfactory Assessment

To ensure functional anosmia amongst the ICA group and normal olfactory function in the control group, olfactory function was assessed in all participants using the Sniffin’ Sticks extended test battery (Burghart Messtechnik GmbH, Holm, Germany) as well as a structured interview with the ICA participants to assess any potential experience of odors, including parental testimony when available. The Sniffin’ Sticks test is a validated and standardized assessment of olfactory ability [21–23] consisting of three subtests measuring odor detection threshold (T), odor quality discrimination (D), and odor quality identification (I), yielding a combined (TDI) score of objective olfactory function (max score of 48). Using normative data from over 9000 people [24], individuals can be classified as either anosmic, hyposmic, or normosmic. In our sample, based on the individual’s TDI score and age, all individuals in the ICA group were classified as anosmic and all individuals in the control group as normosmic.

#### 2.2.2. MRI

Brain imaging data were collected using Siemens Magnetom 3T MR scanners (Siemens Healthcare, Erlangen, Germany). More specifically, two Prisma scanners using 20‐channel head coils and one Verio scanner using a 32‐channel head coil were used. T1‐weighted images covering the entire brain were acquired using either a 3D GR/IR T1‐weighted sequence (208 slices, TR = 2300 ms, TE = 2.89 ms, FA = 9°, voxel size = 1 mm^3^, and FoV = 256 mm) or an MP‐RAGE sequence (176 slices, TR = 1900 ms, TE = 2.52 ms, FA = 9°, voxel size = 1 mm^3^, and FoV = 256 mm).

### 2.3. Analysis

#### 2.3.1. Voxel‐Based Morphometry

To segment T1‐weighted images into gray matter, WM, and cerebrospinal fluid in native space, voxel‐based morphometry [25] was conducted using the SPM12 software. Gray and WM segmented images were fed into the DARTEL toolbox [26] for alignment of the gray and WM of all participants and generation of a brain template through an iterative alignment process. All segmented images and the generated template were then normalized to MNI space using 12‐parameter affine normalization. The resulting images were then modulated by the Jacobian determinants of the deformation fields and smoothed using a 6 mm Gaussian kernel.

Voxel‐wise group differences in WM volume between the ICA and control groups were assessed using an independent‐samples *t*‐test with age, sex, and data collection site as nuisance covariates. A masking threshold of 0.15 was applied to exclude non‐WM voxels. The voxel‐wise group comparison was finally assessed at family‐wise error (FWE)‐corrected *p*  < 0.05.

#### 2.3.2. Searchlight Classification

A searchlight classification approach was applied to investigate whether WM volume patterns could distinguish individuals with ICA from the controls. Using the CoSMoMVPA toolbox [27], we first segmented the WM images into spherical clusters with a 6 mm radius. Each cluster was then analyzed using a linear support vector machine (SVM) classifier trained to classify participants as either ICA or controls. To ensure robust classification, we employed a 10‐fold cross‐validation strategy, training the classifier on 80% of participants and testing it on the remaining 20%, maintaining a balanced distribution across groups. Classification accuracy for each cluster was then mapped onto the center voxel of the corresponding sphere. This process was repeated across the entire brain, resulting in a whole‐brain classification accuracy map. Clusters with classification accuracy greater than 80% were subsequently evaluated for statistical significance. For each cluster, 5000 permutations were performed by randomly shuffling group labels, and observed accuracy exceeding 95% of the accuracy of permuted data was considered significant (*p* < 0.05). Permutation testing was performed only within the high‐accuracy clusters, acknowledging that this cluster‐wise approach does not account for multiple comparisons across the whole‐brain and obtained results, therefore, should be considered exploratory and hypothesis‐generating.

## 3. Results

### 3.1. Orbitofrontal WM Reduction in ICA

**Figure 1:**
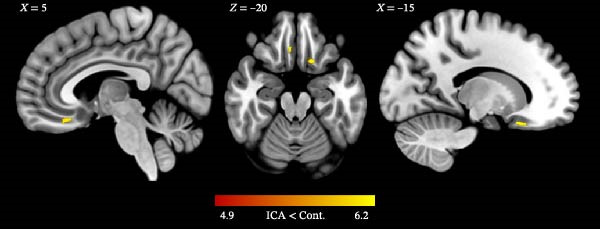
White matter reduction in isolated congenital anosmia (ICA). Individuals with ICA showed reduced white matter volume bilaterally in the orbitofrontal region compared to controls (*p*_FWE-corrected_ <0.009). Colors indicate *t*‐values.

First, we assessed ICA‐related alterations in WM volume with a voxel‐wise analysis on segmented WM maps. We found significantly reduced WM volume in the bilateral orbitofrontal region in ICA individuals compared to control individuals (*p*_FWE-corrected_ <0.009; Figure 1, Table 2). Additionally, we used a more liberal threshold (*p*_uncorrected_ <0.001) to assess potential differences between ICA and control individuals in the piriform cortex but found no significant effect extending to this region.

**Table 2:** Significant areas of difference between ICA and control individuals.

Region	Hemisphere	*x*	*y*	*z*	Cluster size	*T*	*p*_FWE-corrected_
Orbitofrontal	L	−15	20	−23	182	6.14	0.003
Orbitofrontal	R	5	30	−20	88	5.14	0.009

*Note: p*_FWE-corrected_ denotes family wise error corrected *p*‐values.

To account for potential scanner‐ and site‐related variability, the main effect of data collection site was tested within the group comparison model. The observed effect of site was restricted to small regions in the cerebellum and thalamus (Figure S1) and did not overlap spatially with the orbitofrontal clusters showing group differences, indicating that the reported effects are unlikely to be driven by site‐related variance.

### 3.2. Searchlight Classifier Distinguished Between ICA Group and Controls

**Figure 2:**
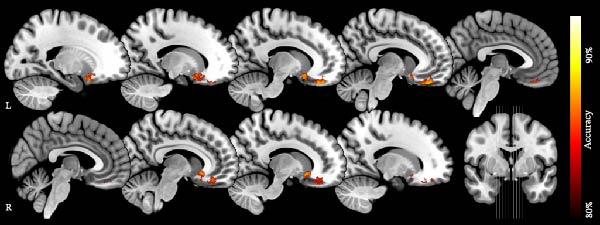
Group classification accuracies between individuals with congenital anosmia and controls, based on white matter volume measures. Significant clusters with accuracy >0.80 are located around orbitofrontal region (*p* < 0.0002). Colors denote percent correct classification between the two groups (accuracy).

Given that voxel‐based assessments indicate differences in individual and independent voxels, we wanted to assess also whether summated changes across neighboring structures would be more sensitive to potential changes. Based on the observed altered WM volume in ICA individuals, we hypothesized that summated WM volume could distinguish ICA individuals from controls. To test this, we applied a searchlight SVM classifier. The classifier identified two clusters (Figure 2, Table 3) with an accuracy threshold >0.80, centered around the bilateral orbitofrontal region that significantly distinguished ICA individuals from normosmic controls. A permutation test confirmed the statistical significance of these clusters (*p* < 0.0002).

**Table 3:** Areas significantly distinguished ICA individuals from controls.

Region	Hemisphere	Cluster size	Peak coordinates (*x*, *y*, *z*)	Peak accuracy
Orbitofrontal	L	2437	−6, 42, −24	0.90
Orbitofrontal	R	1581	8, 20, −9	0.88

## 4. Discussion

We here demonstrate that individuals with ICA have distinct but limited WM alterations as compared to normosmic controls. Specifically, ICA individuals have reduced WM volume, bilaterally, in two clusters of the OFC surrounding the olfactory sulcus, a secondary olfactory cortex, and a central hub for olfactory processing. These findings align with prior work concluding that the volume of this area not only positively predicts olfactory perceptual abilities in healthy controls but is also smaller in those lacking olfactory bulbs from birth [2]. Without olfactory bulb activity, axonal myelination and WM integrity cannot mature normally. This pattern is also observed in studies on individuals with acquired anosmia, where both the OFC and the olfactory bulb show volumetric reductions [16].

More notably, we found no significant alterations in WM tracts near the piriform cortex, the primary sensory cortex for olfaction. These results are consistent with our previous lack of findings of alterations in gray matter [2, 7], where morphological changes are restricted to the OFC, indicating that congenital olfactory sensory deprivation yields limited structural reorganization. If lifelong odor deprivation alters communicative tracts that connect to the piriform cortex, clear changes should be evident in the uncinate fasciculus WM tract. We did not find any difference in WM between ICA individuals and controls with a normal sense of smell in this area, independent of using voxel‐ or network‐based analysis methods, even when using a liberal threshold. However, prior studies have found evidence of higher WM density in the superior longitudinal fasciculus, a longitudinal WM association tract connecting the anterior with the posterior cortical portions, and superior temporal sulcus [19], as well as increased fractional anisotropy in the bilateral OFC [28] among ICA patients. It is not clear why the results differ between studies. The superior longitudinal fasciculus forms part of a broader dorsal fronto‐temporo‐parietal network supporting multisensory integration by relaying information from the superior temporal sulcus. For example, in congenital blindness and deafness, these association networks, and their connecting tracts, often show reorganization, but with substantial between‐subject variability in both structure and function [29, 30]. In congenital anosmia, Peter et al. [2] reported a significant change in superior temporal sulcus in ICA using both surface‐based morphometry and increased neural processing of audiovisual integration [31, 32]. Together, these findings suggest that the superior temporal sulcus and superior longitudinal fasciculus involvement in ICA is real but subtle and potentially behaviorally modulated. Given that we did not use odors, or even mentioned odors or odor processing, to participants, the lack of odor‐related context might play a role in these diverging results. Moreover, WM density (T1‐based) and fractional anisotropy (diffusion‐based) quantify distinct aspects of WM architecture: macrostructural volume vs. microstructural organization. Therefore, reductions in WM density can coexist with unchanged or even increased fractional anisotropy, depending on how fiber coherence and bundle geometry adapt [33].

Contrary to the large‐scale WM changes observed in individuals with lifelong visual and auditory sensory loss [6, 10], our findings suggest that despite lifelong olfactory deprivation, cerebral morphological alterations are limited. In other words, there seems to be no extensive neural reorganization following a lifelong absence of sensory input in the olfactory system. The changes that we do observe around the olfactory sulcus are likely due to the absence of olfactory bulbs in individuals with ICA. The lack of effect in the primary sensory region, taken together with previous findings on gray matter, suggests that sensory deprivation does not affect the olfactory system in the same substantial way as for our other sensory systems [7]. Potentially, the lack of obvious effects could be linked to the fact that the olfactory system differs from other sensory systems in that the primary sensory cortex (the piriform cortex) is involved in more complex processing and consists of a different type of cortical tissue compared to the other primary sensory cortices [34]. In addition, we have recently shown that the piriform cortex also processes also nonolfactory visual and auditory stimuli [35]. If the olfactory system is regularly engaged in processing nonolfactory input, the synaptic pruning mechanism may be absent in the olfactory system of individuals with ICA.

The lack of wide‐range morphological changes also raises questions regarding compensatory mechanisms and whether these exist within the olfactory network or not. Further investigations could help clarify whether individuals with ICA exhibit neural adaptations that compensate for their lack of olfactory input. As outlined above, the fact that the identified morphological changes are limited could potentially be attributed to the olfactory cerebral system being occupied by other functions, such as visual or auditory processing, in individuals suffering from olfactory sensory loss. In line with this argument is our recent demonstration [36] of individuals with congenital anosmia exhibiting greater multisensory processing compared to healthy controls. If one sensory system is absent, alternative pathways may be strengthened and thereby increasing connectivity or WM integrity in other systems or pathways linking sensory areas. In other words, the piriform cortex in ICA individuals may very well process visual and auditory sensory stimuli and could also be more attuned to nonolfactory sensory processing than control individuals with a normal sense of smell [35]. The increased sensory processing in the piriform cortex of visual and auditory stimuli could potentially compensate for the loss of olfactory‐related processing that would have resulted in reduced WM. Alternatively, the olfactory system might be more robust than our other sensory systems against morphological alterations. Speaking against the robustness account are studies indicating that central olfactory cortices in healthy individuals exhibit experience‐dependent plasticity. For example, perceptual learning rapidly updates neural odor representations in the piriform cortex and OFC, with concomitant changes in behavioral discrimination performance [37] to learning, olfactory training, and recovery have been associated with plastic changes within the central olfactory network, including piriform‐centered connectivity changes [38, 39] These findings further align with the fact that also peripheral structures show large neuroplasticity, where, for example, olfactory bulb morphology is altered by both changes in the amount of odor sensations [40] and disease states [41]. Taken together, the available literature suggests that regions within the human olfactory system possess considerable functional and morphological neuroplastic abilities, thereby providing considerable support for the development of compensatory mechanisms.

Further, the olfactory system is also one of the few places where adult neurogenesis has been demonstrated [42]. It is therefore likely that the olfactory system’s involvement in amodal sensory processing (i.e., integration of information not specific to a single sensory modality) is the most convincing explanation of the lack of clear and widespread effects. Further research is warranted to explore the functional implications of these findings and to determine whether olfactory‐related brain regions are repurposed for other cognitive or sensory functions in individuals with olfactory sensory deprivation. Differences in morphological changes related to the onset of sensory deprivation have been established in both vision and audition, with morphological changes varying depending on whether sensory loss is congenital or acquired. With the current sample, we can only establish the effects of a lifelong absence of olfactory input and cannot definitively conclude that the same results would emerge in individuals with acquired smell loss, caused by, for example, viral infections or trauma.

## 5. Conclusion

In conclusion, our study demonstrates that congenital anosmia is associated with subtle but specific WM alterations, primarily localized around the olfactory sulcus. The absence of widespread WM changes suggests that the olfactory system exhibits more limited structural reorganization in response to congenital sensory deprivation than in the visual and auditory systems.

## Author Contributions

Johan N. Lundström contributed to the conceptualization, design, and funding of the study. Moa Peter, Evelina Thunell, and Sanne Boesveldt collected the data. Fahimeh Darki performed the formal analyses. Anja L. Winter wrote the initial draft of the manuscript.

## Funding

Funding was provided by grants awarded to Johan N. Lundström from the Knut and Alice Wallenberg Foundation (Grant KAW 2018.0152) and the Swedish Research Council (Grant 2017‐02325).

## Disclosure

All authors contributed to manuscript revision, as well as read and approved the submitted version.

## Conflicts of Interest

The authors declare no conflicts of interest.

## Supporting Information

Additional supporting information can be found online in the Supporting Information section.

## Supporting information


**Supporting Information** Figure S1 demonstrating how the observed effect of site was restricted to small regions in the cerebellum and thalamus and did not overlap spatially with the orbitofrontal clusters showing group differences, indicating that the reported effects are unlikely to be driven by site‐related variance. Areas indicating white matter reduction in ICA are highlighted in yellow. The effect of site is represented in blue, emphasizing its localized impact within specific regions of the cerebellum and thalamus. Notably, there is no overlap between the yellow and blue regions.

## Data Availability

The data that support the findings of this study are available upon request from the corresponding author. The data are not publicly available due to privacy or ethical restrictions.
