# Supplementary material for: A Life Without Olfactory Function Has Limited Effects on Cerebral White Matter Morphology
Source: Neural Plast. 2026 May 16;2026:3960593. doi: 10.1155/np/3960593 (PMC13179714; doi:10.1155/np/3960593)
Supplement: Supplementary file 1 — Supporting Information Figure S1 demonstrating how the observed effect of site was restricted to small regions in the cerebellum and thalamus and did not overlap spatially with the orbitofrontal clusters showing group differences, indicating that the reported effects are unlikely to be driven by site‐related variance. Areas indicating white matter reduction in ICA are highlighted in yellow. The effect of site is represented in blue, emphasizing its localized impact within specific regions of the cerebellum and thalamus. Notably, there is no overlap between the yellow and blue regions. [file NP-2026-3960593-s001.docx]

**SUPPLEMENTARY MATERIALS**


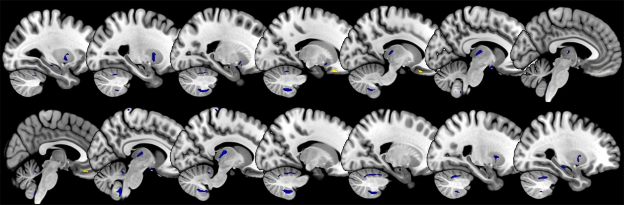
**Supplementary Figure 1.** Effect of data collection site. Areas indicating white matter reduction in ICA are highlighted in yellow. The effect of site is represented in blue, emphasizing its localized impact within specific regions of the cerebellum and thalamus. Notably, there is no overlap between the yellow and blue regions.
